# Supplementary material for: Using single nucleotide variations in single-cell RNA-seq to identify subpopulations and genotype-phenotype linkage
Source: Nat Commun. 2018 Nov 20;9:4892. doi: 10.1038/s41467-018-07170-5 (PMC6244222; doi:10.1038/s41467-018-07170-5)
Supplement: Supplementary file 1 — Supplementary Information [file 41467_2018_7170_MOESM1_ESM.pdf]

Supplementary information for

Using Single Nucleotide Variations in Single-Cell RNA-Seq to Identify Subpopulations and Genotype-phenotype Linkage

Olivier Poirion[1], Xun Zhu[1, 2], Travers Ching[1, 2], Lana X. Garmire [3 *]

[1]Epidemiology Program, University of Hawaii Cancer Center, Honolulu, HI 96813, USA.

[2]Molecular Biosciences and Bioengineering Graduate Program, University of Hawaii at Manoa, Honolulu, HI 96822, USA.

[3]Department of Computational Medicine and Bioinformatics, Building 520, 1600 Huron Parkway, Ann Arbor, MI 48109
[*] To whom correspondence should be addressed. Email address: lgarmire@med.umich.edu

Supplementary Figures

Supplementary Figure 1| SNV calling pipeline.

Supplementary Figure 2| Performance comparison of GATK vs. FreeBayes.

Supplementary Figure 3| SSrGE framework.

Supplementary Figure 4| Simulated SNVs models.

Supplementary Figure 5| Accuracy-regularization relationships.

Supplementary Figure 6| Clustering performance.

Supplementary Figure 7| Pseudo-time reconstruction.

Supplementary Figure 8| Subpopulations from breast cancer patients.

Supplementary Figure 9| Batch-effect comparison.

Supplementary Figure 10| eeSNV inference for nine additional datasets.

Supplementary Figure 11| Minimum spanning trees constructed using HLA features.

Supplementary Table

Supplementary Table 1| Genotype similarity with and without allelic specific expression.

Supplementary Data Files

Supplementary Data File 1| Influence of regularization values for clustering.

Supplementary Data File 2| Ranked features.

Supplementary Data File 3| Highlighted genes for Kim and Chung datasets.

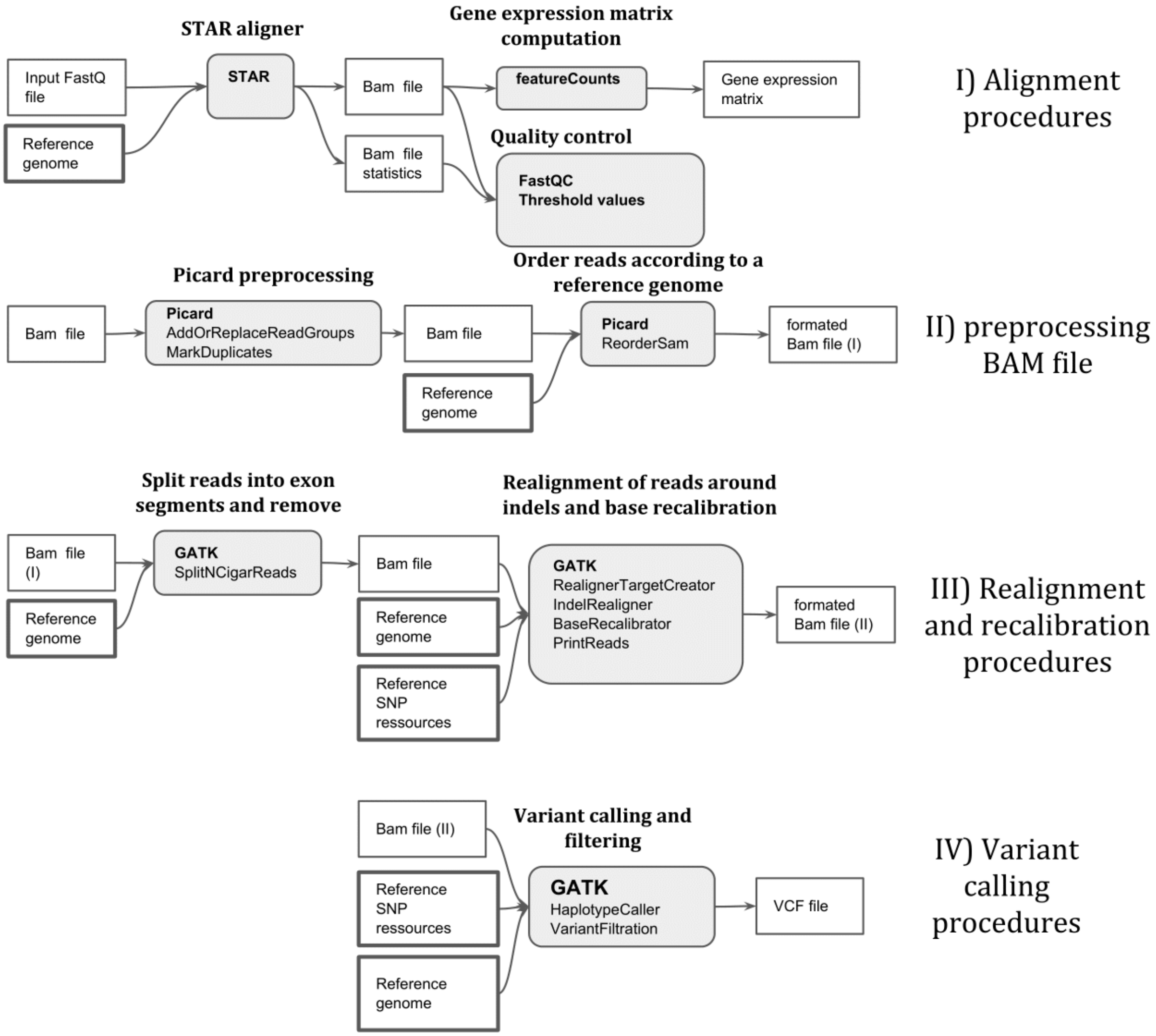

Supplementary Figure 1| SNV calling pipeline. The SNV calling pipeline based on GATK. It follows the best practice workflow for SNP and INDEL calling as recommended, with four steps. Step 1: alignment. Step 2: preprocessing of BAM files. Step 3: read realignment and recalibration. Step 4: variant calling.

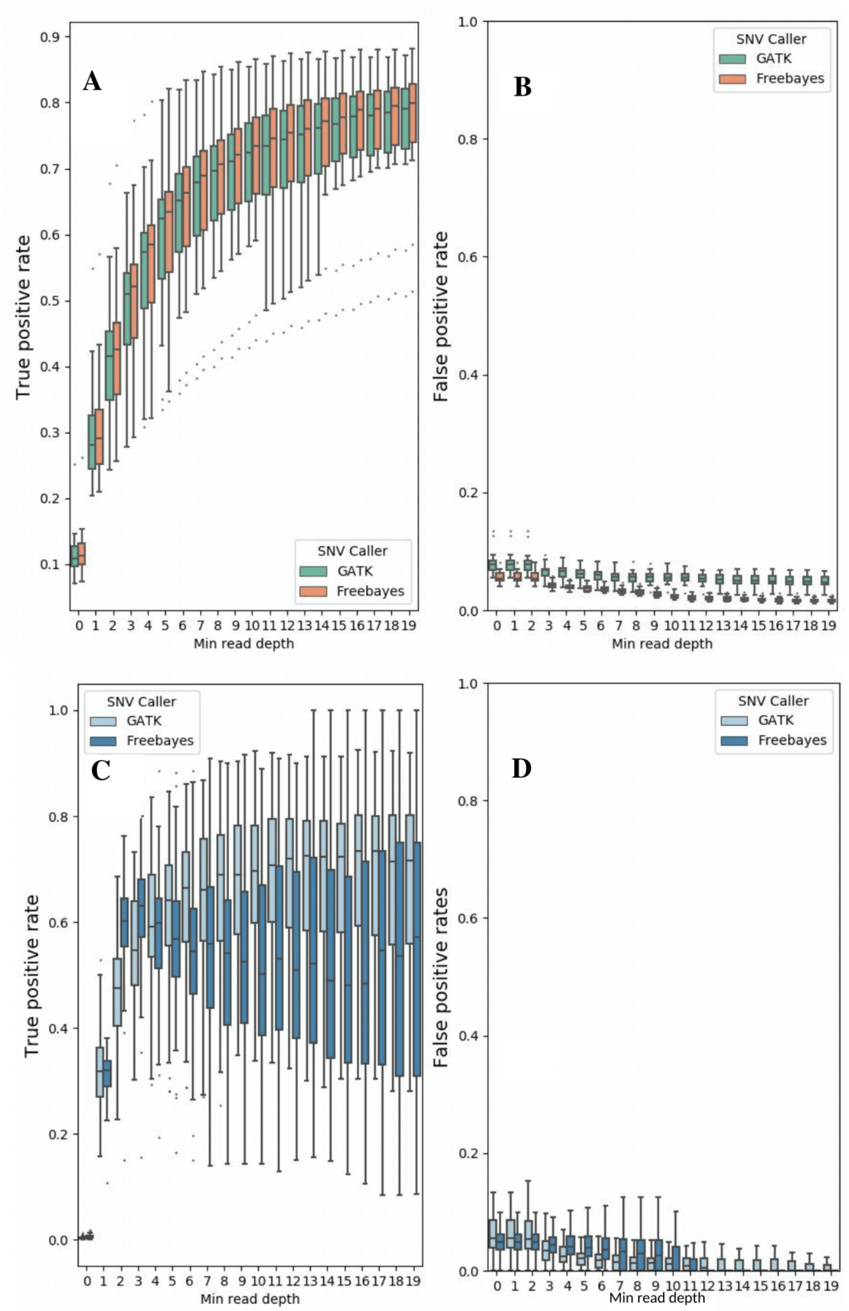

Supplementary Figure 2| Performance comparison of GATK vs. FreeBayes. Performance comparison of GATK vs. FreeBayes SNV calling pipeline using a modified reference h19 genomes containing 50000 random mutations in the exonic region. (A-B) Box plots using a subset of 20 cells from the Kim dataset: (A) true positive rate; (B) false positive rate. (C-D) Box plots using a subset of 100 cells from a 10X genomic dataset: (C) true positive rate; (D) false positive rate. Error bars represent standard deviation from the rates.

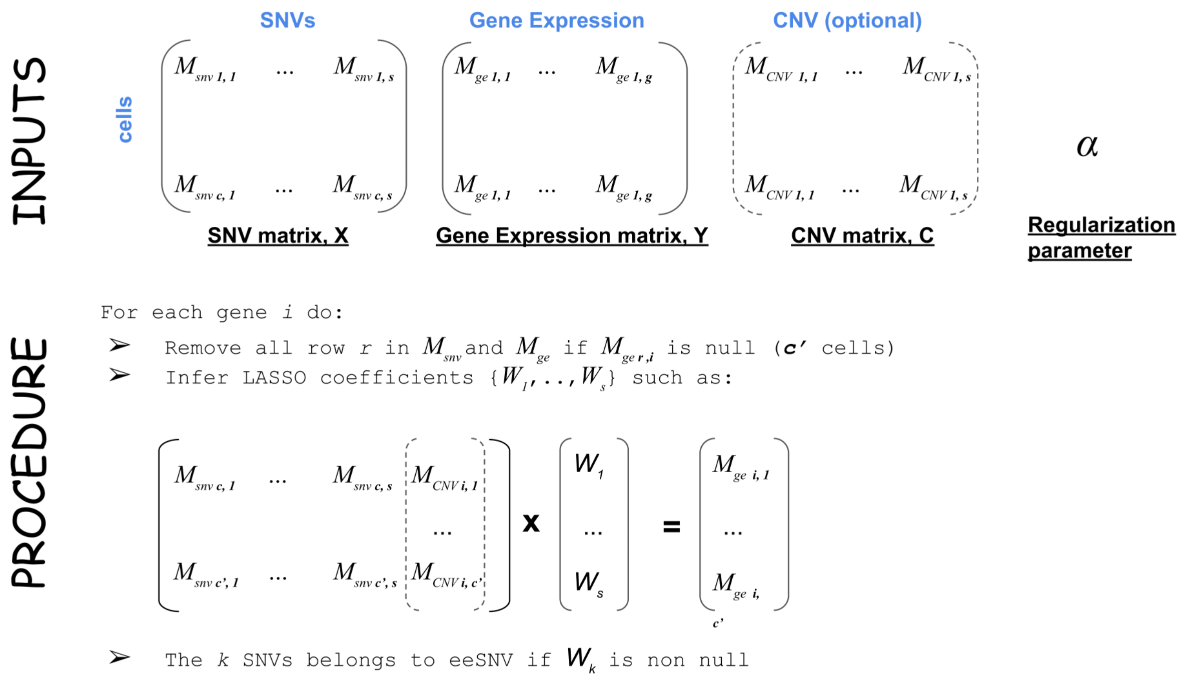

Supplementary Figure 3| SSrGE framework. Sketch of Sparse SNV inference to Reflect Gene Expression (SSrGE) linear models. The SNVs can be calculated from the GATK pipeline (Supplementary Figure 1) or another SNV calling pipeline preferred by users. These SNVs are transformed into a predictor matrix M_{SNV}. As an option, the users can also include a CNV matrix M_{CNV} as an additional predictor matrix. CNVs can be inferred from scRNA-Seq data using programs such as the online platform Ginkgo[69]. Gene expression is the response matrix M_{GE}. For each gene, a LASSO regression is fitted to identify non-null coefficient matrix W. The output of the models is a set of filtered eeSNVs and a set of corresponding genes in which eeSNVs are found.

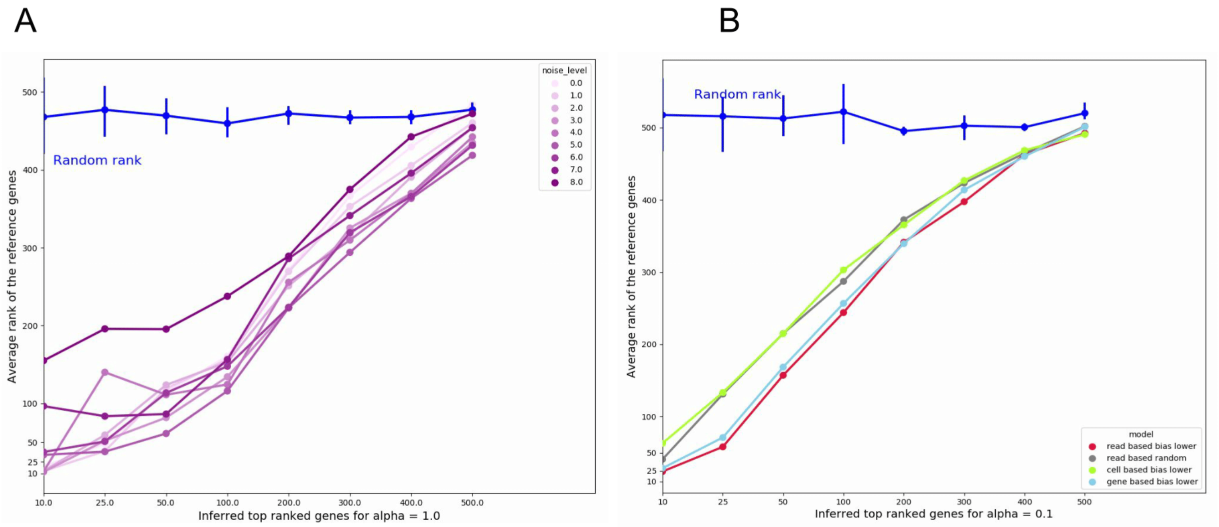

Supplementary Figure 4| Simulated SNVs models. perturbation-model based simulation to evaluate SSrGE quantitatively. Comparisons are performed between the average expected ranks from top genes inferred by SSrGE (x-axis) vs. those set by the simulation (y-axis). (A) Effect of different noise levels. (B) Effect of dropout bias and dropout rate dependency. For dropout bias, it is either biased towards lower expressed genes or random (no bias). Dropout rate is dependent on cell, gene, or reads. Error bars represent standard deviation for a random rank inferred from the corresponding number of genes.

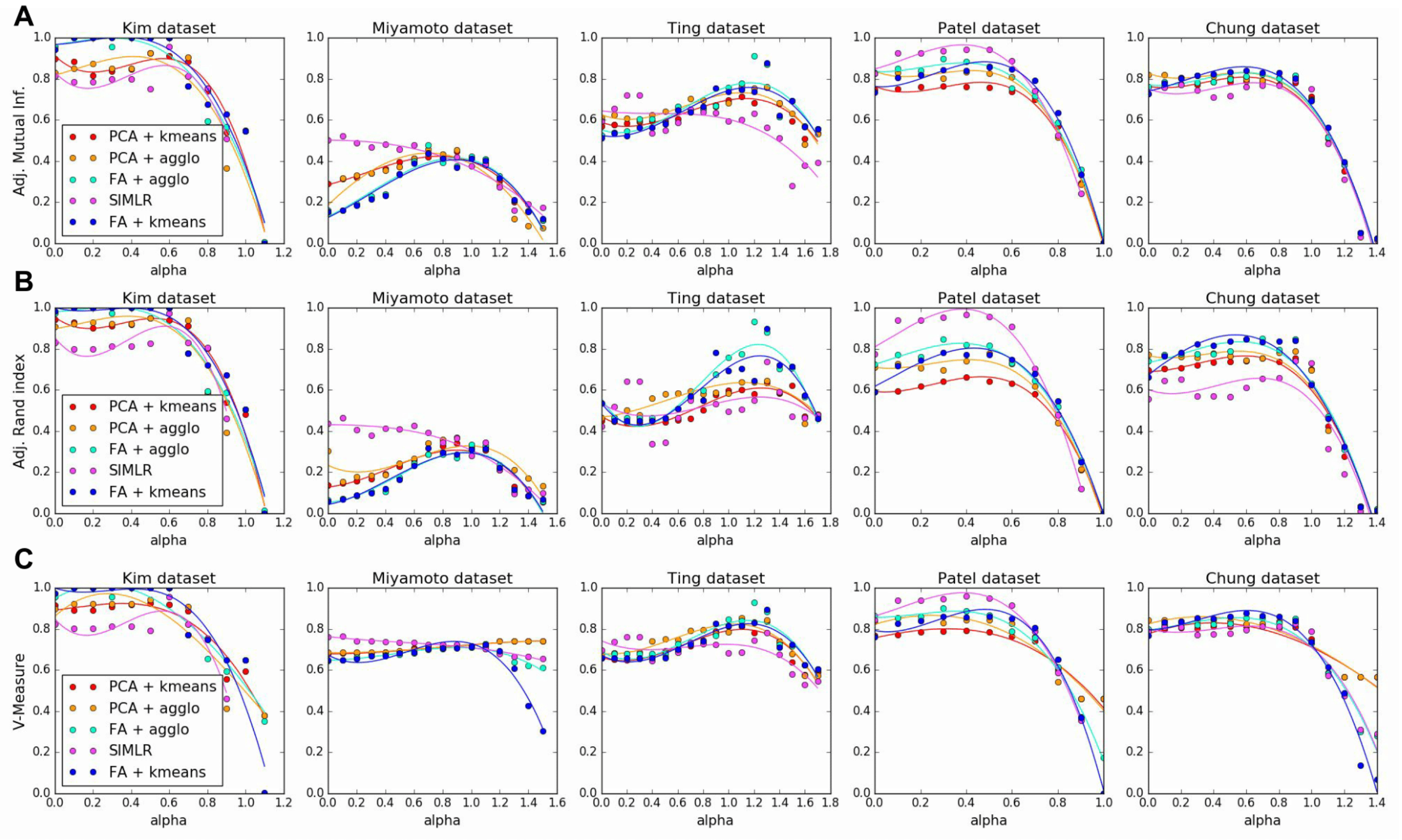

Supplementary Figure 5| Accuracy-regularization relationships. Relationship between the best accuracy metrics and the LASSO regularization parameter *a*, over the five datasets and five different clustering approaches. The accuracy metrics are: (A) Adjusted Mutual Information (AMI), B: Adjusted Rand Index (ARI), and (C): V-measure.

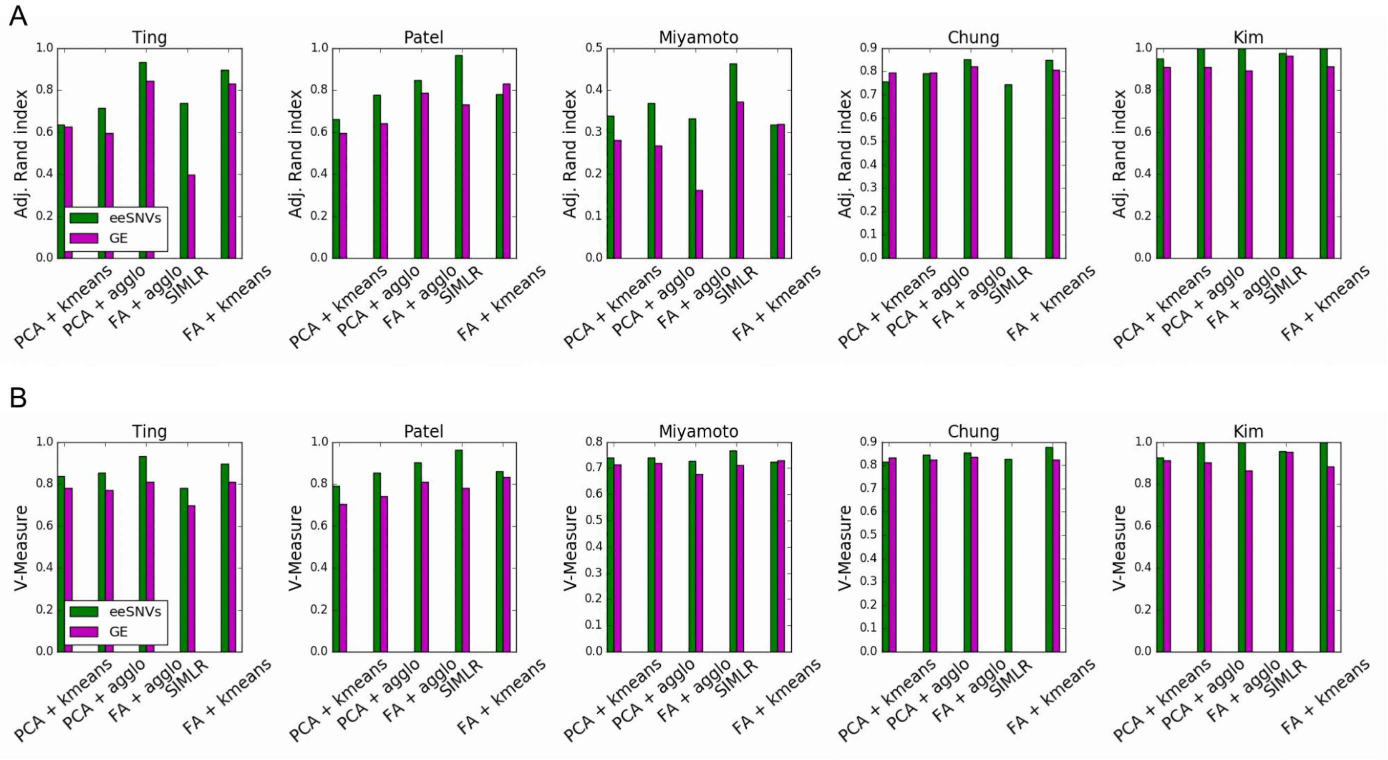

Supplementary Figure 6| Clustering performance. Bar plot comparing the clustering performance using eeSNV vs. gene expression (GE) as features, over five datasets and five different clustering strategies. The metrics used are (A): Adjusted Rand Index (ARI), and (B): V-measure.

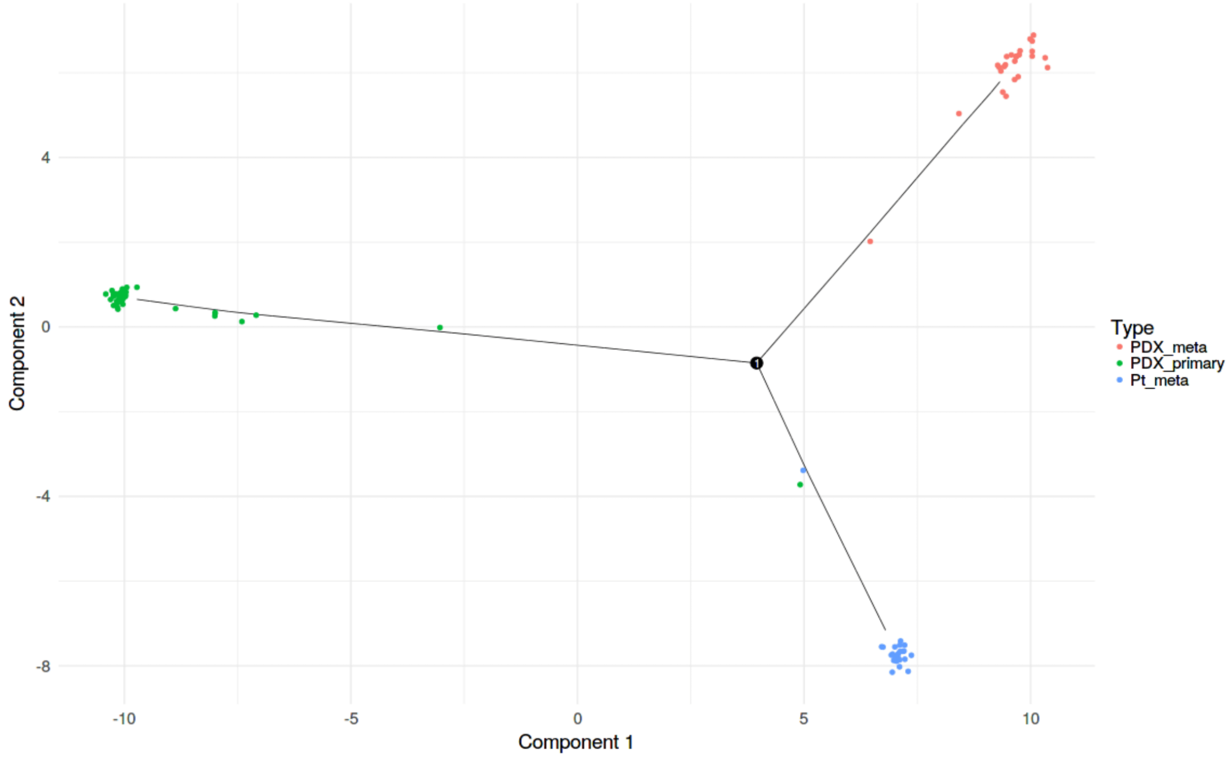

Supplementary Figure 7| Pseudo-time reconstruction. Pseudo-time reconstruction using the Monocle algorithm with gene expression features from genes having eeSNVs, as compared to the pseudo-time reconstruction using eeSNVs in the same genes shown in Figure 6A.

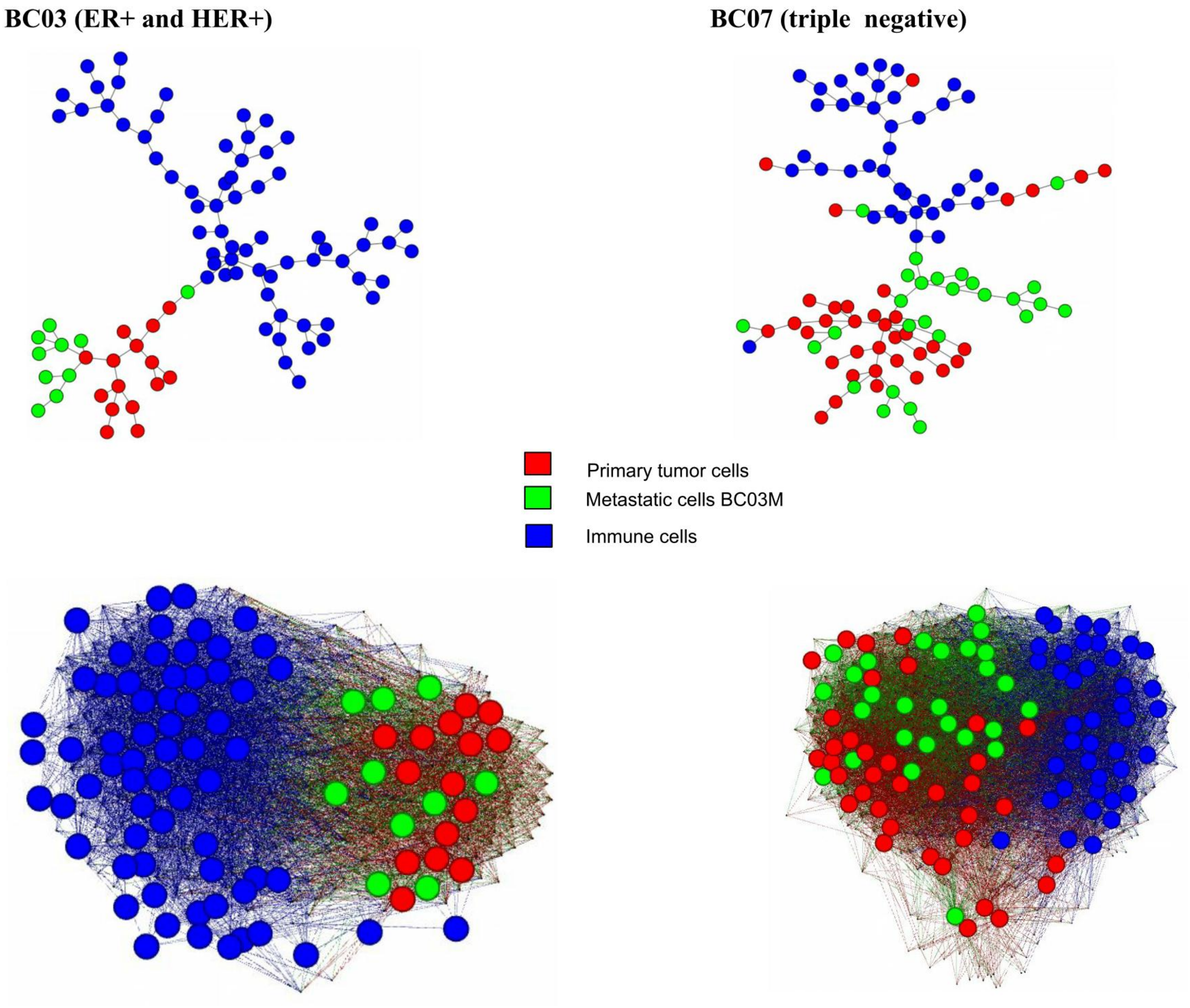

Supplementary Figure 8| Subpopulations from breast cancer patients. Immune (blue), primary (red) and metastatic (green) tumor cell subpopulations from two breast cancer patients (BC03 and BC07) using either bipartite graphs or minimum spanning trees (Chung dataset).

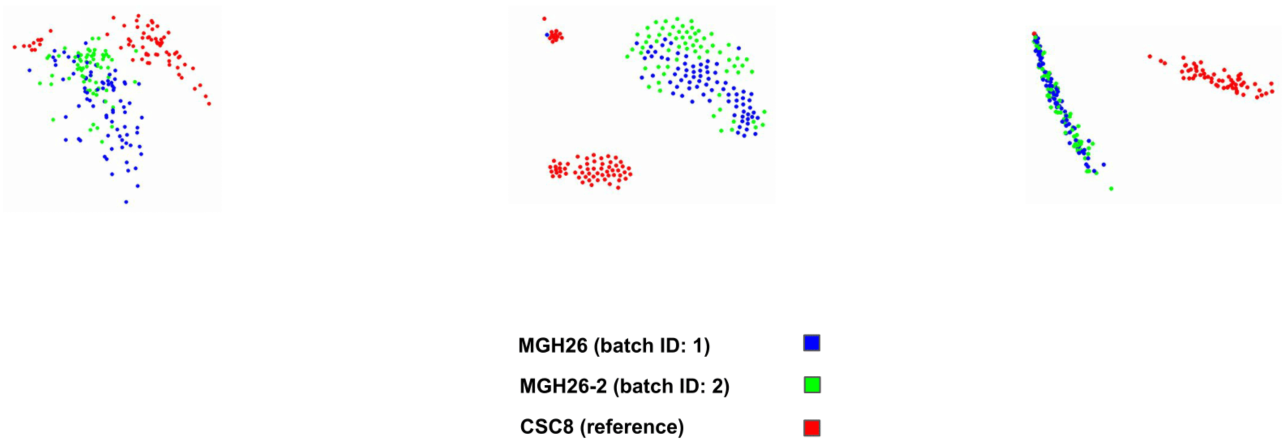

Supplementary Figure 9| Batch-effect comparison. Comparison of the batch-effect on SNVs and gene expression, using scRNA-seq data from glioblastoma patient MGH26.

A

Accession: GSE79457
Title: "Converting adult pancreatic α-cells into β-cells by targeting Dnmt1 and Arx"
Nb. cells: 181
Ref. Genome: Mouse
Remark. Cells after 8 and 12 weeks posterior to insulin related gene shutdown (Dnmt1 and arx shutdown)
Colors: 8 weeks (blue) and 12 weeks (red).
Nb. of eeSNvs: 425
Top 8 ranked genes: Gcg, Rn45s, Pisd-ps2, Pisd-ps1, Ccnd2, Fam20c, Ndufs2, Ddc

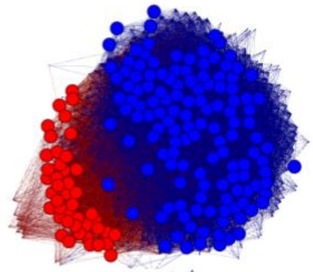

B

Accession: GSE81383
Title: "Mapping heterogeneity in a patient-derived melanoma culture by single-cell RNA-seq"
Nb. cells: 301
Ref. genome: Human
Cells: Single-cells from 3 different melanoma patients with 3 distinct melanoma genotypes
Colors: BRAF V600E mutant and NRAS wild type (red), BRAF wild type and NRAS G13R mutant (green), and double negative (blue)
Nb. of eeSNvs: 275
Top 8 ranked genes: FKBP1A, TIMP3, VIM, ZNF17, IRF4, YWHAE, RPL14, ALG11

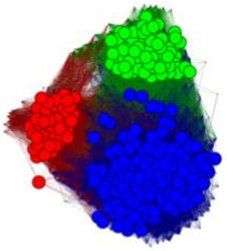

C

Accession: GSE69405
Title: "Single-cell RNA sequencing of lung adenocarcinoma patient-derived cells"
Nb. cells: 180
Ref. genome: Human PDX
Colors: H358 human lung cancer cells (red), Lung cancer PDX case 1 (yellow) and Lung cancer case 2 from primary (blue and cyan for the replicate) tumor
Nb. of eeSNvs: 458
Top 8 ranked genes: ACTG1, CEACAM6, SCP2, GPNMB, TTC39C, HLA-DQB1, BACH1, IPO9

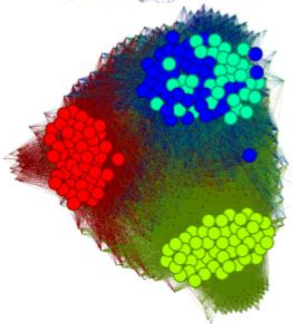

D

Accession: GSE81903
Title: "Smart-seq2 analysis of P17 FACS sorted retinal cells from the Kcng4-cre;stop-YFP X Thy1-stop-YFP Line"
Nb. cells: 137
Ref. genome: Mouse
Cells: Four mouse retinas (1la, 1ra, 2la, and 2ra) from two mices
Colors: 1la (green) and 1lr (red) retina from mouse 1 and 2la (blue) and 2ra (cyan) retina from mouse 2
Nb. of eeSNvs: 293
Top 8 ranked genes: Rn45s, Pisd-ps1, Dstn, Snap25, Isl1, Tcf25, Reln, Rpa1

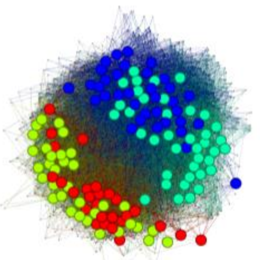

E

Accession: Frozen bmmc healthy donors 1 and 2 from https://support.10xgenomics.com/single-cell-gene-expression/datasets
Nb. cells: 1985
Ref. genome: Human
Colors: Healthy donor 1 in Blue and Healthy donor 2 in red
Remark: Combination of 2 10XGenomics datasets from 2 distinct healthy patients. We downloaded the two datasets separately and used 1000 cells from each patient together to generate the input fastq files
Nb. of eeSNvs: 439
Top 8 ranked genes: AHSP HLA-B, HBA2, LRRC75A-AS1, HLA-DRA, GLRX5, GYPC, HLA-C

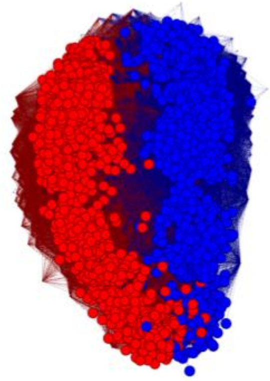

F

Accession: GSE80232
Title: "Smart-seq2 analysis of P17 FACS sorted retinal cells from the Tg(Chx10-EGFP/cre,-ALPP)2Clc or Vsx2-GFP transgenic line"
Nb. cells: 255
Ref. genome: Mouse
Cells: three mouse retinas (1a, 2a, 2b)
Colors: 1a (red), 2a (green) and 2b (blue). 2a and 2b are most likely from the same mouse
Nb. of eeSNvs: 539
Top 8 ranked genes: Pdpn, Rn45s, 2700089E24Rik, Lactbl1, Snap25, E130218I03Rik, Rtn1, Atp6v1b2

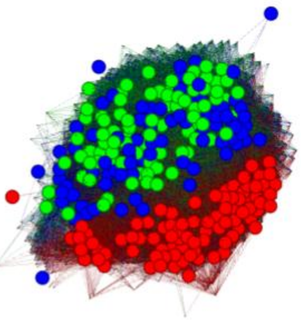

G

Accession: https://support.10xgenomics.com/single-cell-gene-expression/datasets/1.1.0/frozen_pbmc_b_c_50_50
Nb. cells: 3291
Ref. genome: Human
Cells: Single-cells from two PBMC donors and generated using 10X Genomics
Remark: Non cancer 10XGenomics dataset. We collected the fastq files of 3300 cells from the original dataset. The original analysis highlighted different subpopulations based on GE using either tSNE, or K-Means. (see http://cf.10xgenomics.com/samples/cell-exp/1.1.0/frozen_pbmc_b_c_50_50/frozen_pbmc_b_c_50_50_web_summary.html). Here, the graph identifies two distinct clusters that likely corresponds to the cells of the two donors. In addition, It seems that different subgroups exist inside each cluster that might correspond to different cell populations.
Colors: (G.a) K-Means (K=4) clusters found in the original analysis (G.b) clusters extracted from the eeSNVs graph
Nb. of eeSNvs: 678
Top 8 ranked genes: HLA-DRA, HLA-DRB1, CYBA, PSMA7, GAPDH, RPL13, HLA-DRB5, RPS4X

G.a

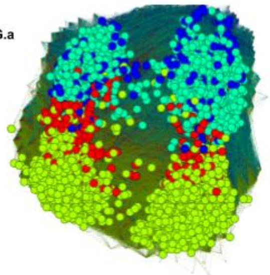

G.b

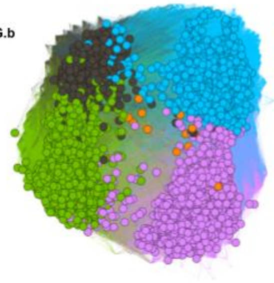

H

Accession: GSE65253
Title.: "Prospective derivation of a 'Living Organoid Biobank' of colorectal cancer patients"
Nb. cells: 108
Ref. genome: Human
Cells: single cells from patient organoids
Remark: Only 14 cells passed the quality filter
Colors: 8 different patients are represented each with a distinct color
Nb. of eeSNvs: 504
Top 8 ranked genes: PLEC , HSP90AA1, CNN2, LRRC47, LAMP1, CDC27

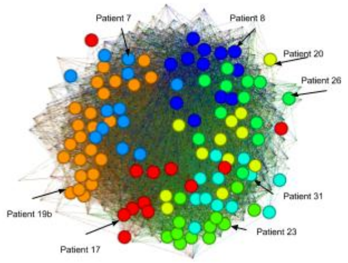

I

Accession: GSE71485
Title: "Single-cell RNA-seq with Waterfall Reveals Molecular Cascades underlying Adult Hippocampal Neurogenesis"
Nb. cells: 85
Ref. genome: Mouse
Cells: Time serie of mouse hippocampal differentiation from 8 to 12 weeks
Remark: Non cancer
Colors: Differentiation stage from early (blue) to late (red)
Nb. of eeSNvs: 968
Top 8 ranked genes: Rn45s, Msi2, Son, Sox9, Slc1a2, Pisd-ps1, Tcf4

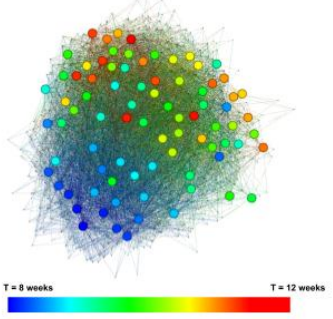

Supplementary Figure 10| eeSNV inference for nine additional datasets. eeSNV inference, gene ranking, and cell visualization for 9 additional datasets. Datasets E and G are 10X Genomic datasets from the 10X Genomic database website: https://support.10xgenomics.com/single-cell-gene-expression. The other datasets A, B, C, D, F, H, and I are extracted from NCBI GEO. The top 8 genes are presented for each dataset, based on their eeSNV scores.

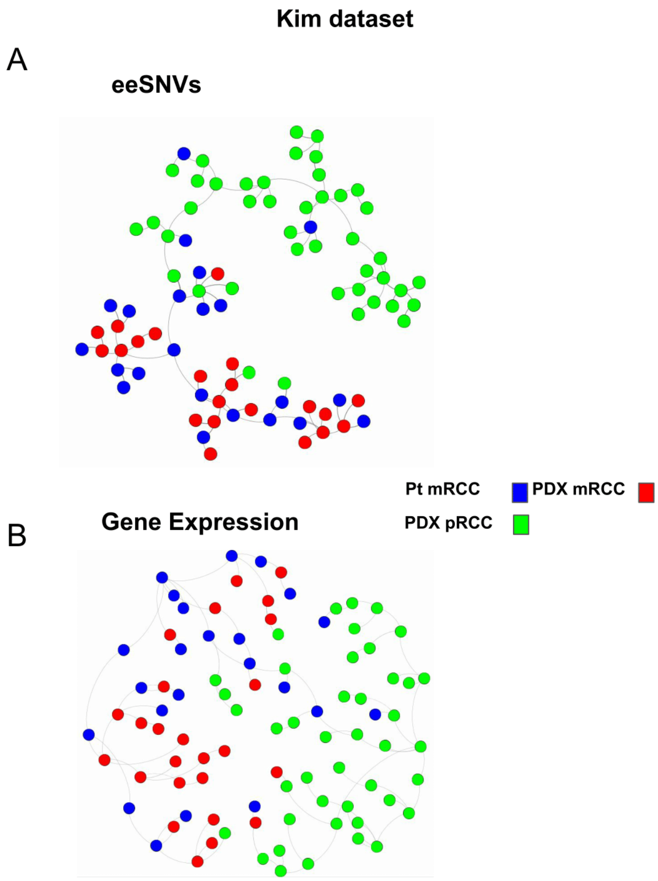

Supplementary Figure 11| Minimum spanning trees constructed using HLA features. Minimum Spanning tree using either eeSNVs (A) or gene expression (B) of HLA as features. Pearson correlation is used as the distance metric. Color labels: primary tumor (green), metastatic cells from patients (blue) and metastatic cells from patient derived xenografts (red).

Supplementary Table 1| Genotype similarity with and without allelic specific expression. Genotype of SNVs and similarity testing results with and without allelic specific expression. Average per-cell genotypes of the SNVs are detected and computed using QuASAR. *g0*, *g1* and *g2* correspond to the *homozygous reference*, *heterozygous,* and *homozygous alternate* genotypes, respectively.

	ranked genes (alpha=0.1)		ranked eeSNVs (alpha=0.1)		Number of eeSNVs per genotype			
**Dataset**	**Kendall Tau score**	**Kendall Tau p-value**	**Kendall Tau score**	**Kendall Tau p-value**	**g0 (homo ref)**	**g1 (heter)**	**g2 (homo alter)**	**Without specific allelic expression**
Kim	0.71	0	0.58	0	1994	4314	11876	13939
Patel	0.73	0	0.62	0	372	2020	6660	7168
Ting	0.64	0	0.53	0	248	2016	14214	15140
Miyamoto	0.68	0	0.58	0	101	1186	4981	5327
Chung	0.76	0	0.53	0	3026	6391	16093	18741

Supplementary Data File 1| Influence of regularization values for clustering. Regularization values (*a*) used for the clustering procedures along with the number of eeSNVs features.

Supplementary Data File 2| Ranked features. Ranked eeSNVs and genes for each dataset (with minimum regularization filtering *a*=0.1).

Supplementary Data File 3| Highlighted genes for Kim and Chung datasets. Ranked genes for the metastasis single-cells from the Kim dataset (mRCC) and from BC03M and BC07M from Chung datasets.
