# Supplementary material for: Using single nucleotide variations in single-cell RNA-seq to identify subpopulations and genotype-phenotype linkage
Source: Nat Commun. 2018 Nov 20;9:4892. doi: 10.1038/s41467-018-07170-5 (PMC6244222; doi:10.1038/s41467-018-07170-5)
Supplement: Supplementary file 2 — Description of Additional Supplementary Files [file 41467_2018_7170_MOESM2_ESM.pdf]

Description of Additional Supplementary Files

File Name: Supplementary Data 1
Description: Influence of regularization values for clustering.

File Name: Supplementary Data 2
Description: Ranked features.

File Name: Supplementary Data 3
Description: Highlighted genes for Kim and Chung datasets.
